# Self-rated health does not predict 10-year weight change among middle-aged adults in a longitudinal population study

**DOI:** 10.1186/1471-2458-11-748

**Published:** 2011-09-30

**Authors:** Margareta Norberg, Kristina Lindvall, Paul L Jenkins, Maria Emmelin, Göran Lönnberg, Anne N Nafziger

**Affiliations:** 1 Department of Public Health and Clinical Medicine, Epidemiology and Global Health, Umeå University, S-901 87 Umeå, Sweden; 2 Department of Public Health and Clinical Medicine, Umeå Centre for Global Health Research, Umeå University, S-901 87 Umeå, Sweden; 3 Centre for Population Studies, Ageing and Living Conditions Programme, Umeå University, S-901 87 Umeå, Sweden; 4 The Research Institute, Bassett Healthcare, One Atwell Road, Cooperstown, NY 13326, USA; 5 New York Center for Agricultural Medicine and Health, Bassett Healthcare, One Atwell Road, Cooperstown, NY 13326, USA; 6 Department of Clinical Sciences, Social Medicine and Global Health, Lund University, Box 117, S-221 00 Lund, Sweden; 7 Bertino Consulting, 3078 New Williamsburg Drive, Schenectady, NY 12303, USA

## Abstract

**Background:**

There is a worldwide obesity epidemic, but lack of a simple method, applicable for research or clinical use, to identify individuals at high risk of weight gain. Therefore, the relationship of self-rated health and 10-year percent weight change was evaluated to determine if self-rated health would predict weight change.

**Methods:**

From 1990 to 2008, adults aged 30, 40, 50 and 60 years were invited to health surveys that included self-rated health and measured weight and height. ANOVA was used to evaluate the relationship of 10-year percent weight change and self-rated health.

**Results:**

The study population consisted of 29,207 participants (46.5% men). There was no relationship between baseline self-rated health and 10-year percent weight change for middle-aged men or women.

**Conclusions:**

Self-rated health is not able to predict weight change over a 10-year period in this age group.

## Background

The obesity epidemic is occurring in many regions and countries of the world. A dose-response relationship exists between increasing obesity and an increasing burden of health problems, mainly cardiovascular diseases, diabetes and cancers [1]. There are also important social and psychological effects of obesity [2,3]. Most of the increasing number of efforts to counteract the obesity epidemic focus on promotion of weight loss and have shown limited or no long-term success [4]. Some work has been done to identify those at greatest risk for weight gain with the idea that interventions targeted at such individuals might be the most cost-effective [5]. More recently there has been increasing interest in seeking to identify those who will maintain body weight, because weight maintenance may be easier to achieve than weight loss with subsequent weight stability [4,6]. Also, primary prevention of weight gain may have the greatest chance of avoiding the morbidity and mortality associated with excess body weight [7].

Self-rated health is known to both predict and be associated with mortality [8] and many health conditions [2,9-13], and to mainly reflect mental and physical health status [14]. However, not all studies have found a consistent association between poor self-rated health and obesity [2,15,16]. Some investigators have found that associations of poor self-rated health are lowest among overweight middle-aged men and normal weight women below 46 years of age [17], while others have found the strongest relationship among middle-aged men [18]. One study found that overweight or obese adults were more than twice as likely to have self-rated poor health [16]. Another study of female nurses (aged 44-69) found that those with normal weight and poor self-rated health are at higher risk of later weight gain than obese nurses [19]. Among adults the relationship of self-rated health to body weight is inconsistent for different age groups and for men versus women [17]. Few data exist about whether self-rated health is a predictor of future weight change or obesity.

We were interested in determining whether self-rated health would predict weight maintenance or change in a middle-aged adult population. If so, this simple question could be used quickly, easily, and inexpensively in a clinical or public health setting. Using a large population-based study, we evaluated 10-year weight change and determined if self-rated health at baseline could predict weight change.

## Methods

### Setting and study population

Västerbotten County is located in Northern Sweden and covers one eighth of the country. The Västerbotten Intervention Programme (VIP) has previously been described in detail [20]. VIP was initiated in 1985 to reduce cardiovascular disease (CVD) mortality rates in Västerbotten County, and successively implemented throughout the county by 1991. All county inhabitants are invited to a health examination the year they turn 40, 50 or 60 years old. Initially, 30 year olds were also invited, but due to low participation rates and economic restrictions, they were not invited after 1995. The cross-sectional study participation rates were 48-57% during 1990-1995 and thereafter increased and have remained at 66-67% since 2005. Participation rates in the 10-year follow-up surveys were 63-72% during 2000-2005 and have remained at 75-79% since 2005.

Surveys consist of questionnaires and health examinations administered by trained district nurses and include assessment of traditional CVD risk markers and a comprehensive questionnaire regarding demographics, socioeconomic status, self-rated health and lifestyle habits. The results of the examination are individually discussed with each participant by a trained district nurse at the end of the survey using motivational interviewing principles. Health promotion activities are also suggested at that time.

This report is based on participants who were aged 30, 40 or 50 years at baseline and were initially surveyed during 1990-1998. Each participant must have returned for a follow-up visit 10 years later (2000-2008) and have valid weight, height and self-rated health data. Individuals gave informed consent prior to each health screening. The study was conducted in compliance with the Helsinki Declaration and was approved by the regional Research Ethics Board in Umeå (08-131M).

### Measurements

Self-rated health was based on the question: "How do you assess your general health during the previous year?" There were five alternatives: "very good", "pretty good", "somewhat good", "pretty bad" and "bad" [21]. Assessment of socioeconomic status included marital status (married/partnership vs. single/divorced), education level (basic <10 yr, mid-level 10-12 yr, or high >12 yr) and type of geographic residence (city [Umeå], small town [Skellefteå or Lycksele], or rural [smaller municipalities]). Family history of CVD and/or diabetes was defined as having answered yes to at least one of the following questions: "Do any of your parents or siblings have a history of myocardial infarction or stroke?" and "Do any of your parents or siblings have a history of diabetes mellitus?" Tobacco habits were categorized based on questions regarding smoking and the use of Swedish oral moist tobacco (snus). Cigarette smoking was categorized into never smokers, ex-smokers and current smokers. Snus use was categorized into non-users and current users. Physical activity was defined as sedentary, moderate activity or physically active, based on leisure time activity and mode of transportation to work.

Examinations were done in the morning after an overnight fast. Height and weight were measured in light indoor clothing. Ten-year percent weight change was calculated as the difference between body weight at 10-year follow-up and baseline weight, divided by baseline weight, and multiplied by 100. Body mass index (BMI, kg/m^2^) was calculated. Capillary glucose and total cholesterol were measured using a Reflotron^® ^analyzer (Boehringer Mannheim GmbH, Mannheim, Germany). An oral glucose tolerance test was offered to all participants not known to have diabetes and with a fasting plasma glucose <7.0 mmol/L [22]. Diabetes was defined as a fasting capillary plasma glucose ≥7.0 mmol/L, or a capillary 2-hour plasma glucose ≥12.2 mmol/L, or having answered "yes" to the question "Do you have diabetes?" Recumbent blood pressure was measured with a mercury sphygmomanometer after 5 minutes of rest.

### Statistics

All statistical analyses were performed with SPSS Statistics version 17.0. Ten-year percent weight change was the outcome of interest. Separate analyses were performed for men and women. A drop-out analysis was done with comparison of characteristics at base-line for participants and non-participants to 10-year follow-up. Because this was a longitudinal data analysis, only follow-up participants were further analysed. Base-line characteristics are given as distributions for categorical variables and mean ± standard deviation for continuous variables.

The relationship between percent weight change and categorical variables was tested by ANOVA and Spearman correlation coefficients. Graphic illustration of 10-year weight change by self-rated health categories was done with box-plot analyses.

One by five analysis of variance (ANOVA) was done for 10-year percent weight change and other continuous variables by self-rated health. Post hoc testing of ANOVA was done with the Scheffe test. Comparison of percent weight change to continuous variables was also done by visual assessment of graphic distributions and Pearson correlation coefficients. Comparison of self-rated health and categorical variables was done with Χ^2 ^testing and the relation between self-rated health and continuous variables with Student's t-test or ANOVA as appropriate.

## Results

Of the 40,555 individuals who participated in VIP from 1990 through 1998, 39,723 were eligible for the 10-year follow-up. A total of 2920 individuals were ineligible for follow-up because 1854 had moved from the region, 1007 had died (2.5% during 10 years), and 59 were excluded for another reason (such as change in person number). Of those eligible, 30,168 (75.9%) participated in the follow-up survey; of these, 46.6% were men and 53.4% were women. Table 1 shows the baseline characteristics of those who participated in the follow-up survey versus the non-respondents.

**Table 1:** Baseline demographic and socioeconomic characteristics and BMI of the study population by participant status at the 10-year follow-up survey

Baseline characteristic	Participants	Non-participants
	n = 30,168	n = 9555

Sex (%)		
Male	46.6	50.4
Female	53.4	49.6%

Age (%)		
30 years old	17.3	23.8
40 years old	40.6	40.3
50 years old	41.6	35.8

Education (%)		
Basic	22.2	22.5
Mid-level	53.6	52.0
High	24.2	25.5

BMI (kg/m^2^), mean ± SD	25.0 ± 3.7	25.5 ± 4.2

Self-rated health (%)		
Very good	29.8	27.8
Pretty good	48.2	47.7
Somewhat good	17.1	18.9
Pretty bad	3.9	4.5
Bad	0.9	1.2

Physical activity (%)		
Sedentary	18.4	19.2
Moderately active	67.9	66.3
Physically active	13.6	14.5

Data are from the baseline visit and presented as percentage or mean ± standard deviation.

Participants who were missing values for weight at baseline or the follow-up survey, baseline height, or self-rated health at either survey were excluded (n = 961), leaving 29,207 participants. Table 2 provides demographic and descriptive characteristics of the study population. Seventy-eight percent reported very good or pretty good self-rated health and less than 5% pretty bad or bad self-rated health. Average 10-year weight change was 4.7% among men and 5.5% among women, and both sexes on average increased just over one BMI-unit during the 10-year period, from a baseline BMI of 25.5 for men and 24.6 for women. One fourth of the population were smokers and the use of snus was more prevalent than smoking among men.

**Table 2:** Demographic, socioeconomic and biologic characteristics of the study population derived from the Västerbotten Intervention Programme surveys

	Men	Women
Number of participants, n (%)	13,583 (46.5)	15,624 (53.5)

Age group, n (%)		
30 yr	2,487 (18.3)	2,698 (17.3)
40 yr	5,482 (40.4)	6,407 (41.0)
50 yr	5,614 (41.3)	6,519 (41.7)

Marital status, n (%)		
Married	11,048 (81.9)	13,304 (85.6)
Not married	2,438 (18.1)	2,245 (14.4)

Education level, n (%)		
Basic	2,922 (21.6)	3,121 (20.1)
Mid-level	7,893 (58.4)	8,045 (51.8)
High	2,689 (19.9)	4,376 (28.2)

Geographic residence, n (%)		
City	5,152 (37.9)	6,253 (40.0)
Small town	4,307 (31.7)	5,068 (32.4)
Rural	4,124 (30.4)	4,303 (27.5)

Family history of CVD or diabetes, n (%)	4,674 (34.7)	6,086 (39.4)

Self-rated health, n (%)		
Very good	4,068 (29.9)	4,647 (29.7)
Pretty good	6,701 (49.3)	7,392 (47.3)
Somewhat good	2,313 (17.0)	2,690 (17.2)
Pretty bad	408 (3.0)	719 (4.6)
Bad	93 (0.7)	176 (1.1)

Physical activity level, n (%)		
Sedentary	3,219 (23.7)	2,135 (13.7)
Moderate activity	8,292 (61.2)	11,551 (74.0)
High activity	2,050 (15.1)	1,915 (12.3)

Weight, kg		
Baseline	81.5 ± 11.7	66.9 ± 11.4
10-year follow-up	85.3 ± 13.0	70.4 ± 12.6

Height at baseline, cm	178.7 ± 6.5	165.1 ± 5.8

Body mass index (kg/m^2^)		
Baseline	25.5 ± 3.3	24.6 ± 4.0
10 year follow up	26.7 ± 3.7	25.9 ± 4.5

10-year percent weight change	4.7 ± 8.2	5.5 ± 9.7

Systolic blood pressure, mmHg	125.5 ± 13.9	120.7 ± 15.3

Diastolic blood pressure, mmHg	78.8 ± 10.1	75.6 ± 10.2

Serum cholesterol, mmol/L	5.7 ± 1.2	5.4 ± 1.1

Fasting capillary glucose, mmol/L	5.3 ± 0.8	5.2 ± 0.7

Diabetes type 2 (WHO criteria), n (%)	534 (4.2)	886 (6.1)

Tobacco use, n (%)		
Current smoker	2,941 (21.9)	4,048 (26.2)
Current snus user	3,516 (26.4)	590 (3.9)

Data are from the baseline visit unless otherwise noted and presented as percentage or mean ± standard deviation.

All continuous and categorical variables except for age group differed between men and women by *p *< 0.001.

**Figure 1:**
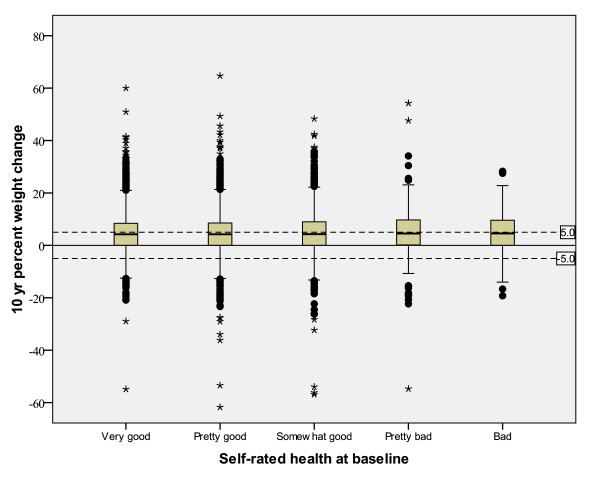
**Ten-year percent weight change and baseline self-rated health (1990-1998) among 13,583 men in the Västerbotten Intervention Programme surveys**. The solid horizontal line is set at 0% weight change. The dashed lines denote -5% and +5% weight change.

**Figure 2:**
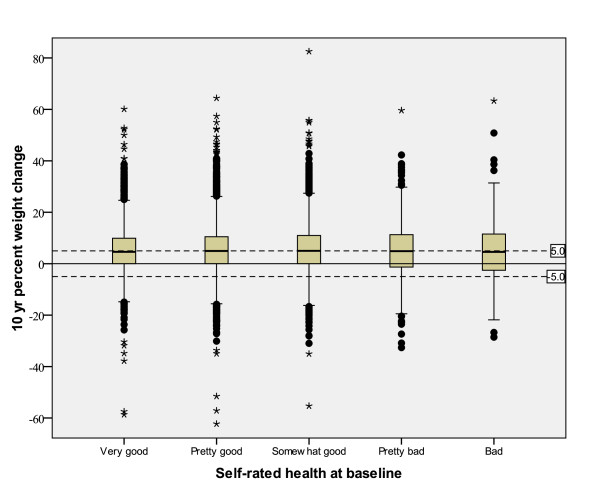
**Ten-year percent weight change and baseline self-rated health (1990-1998) among 15,624 women in the Västerbotten Intervention Programme surveys**. The solid horizontal line is set at 0% weight change. The dashed lines denote -5% and +5% weight change.

Univariate analyses of relationships between 10-year weight change with self-rated health and other categorical variables are shown in Table 3. There was no relationship between self-rated health at baseline and 10-year percent weight change for either men or women. This finding is further supported by weak correlations between self-rated health and 10-year percent weight change of r = 0.004 (*p *= 0.65) for men and r = 0.013 (*p *= 0.11) for women. Box plots illustrate the results (Figures 1 and 2). The means and variability in 10-year percent weight change were similar among the self-rated health groups for both sexes. In complimentary analyses with stratification for smoking, there were no significant relations between self-rated health at baseline and 10-year weigh change in any smoking category in either sex (data not shown). Univariate analyses of relationships between weight change and other biologic variables were all significant in the negative direction (Table 4).

**Table 3:** Mean 10-year percent weight change for categorical variables by sex

	Men		Women	
Variable	(mean ± SD)	*P *value	(mean ± SD)	*P *value

Self-rated health		0.818		0.332
Very good	4.8 ± 8.2		5.2 ± 9.2	
Pretty good	4.7 ± 7.9		5.5 ± 9.3	
Somewhat good	4.8 ± 8.8		5.7 ± 10.4	
Pretty bad	5.0 ± 9.0		5.5 ± 12.5	
Bad	5.9 ± 9.1		5.2 ± 12.7	

Age group		<0.001		<0.001
30 years	7.2 ± 9.3		7.4 ± 11.2	
40 years	5.4 ± 7.7		6.0 ± 9.5	
50 years	3.0 ± 7.7		4.1 ± 8.9	

Marital status		<0.001		<0.001
Married	4.5 ± 7.8		5.2 ± 9.4	
Not married	5.7 ± 9.9		6.8 ± 11.1	

Education level		<0.001		<0.001
Basic	4.0 ± 7.8		5.1 ± 9.9	
Mid-level	5.1 ± 8.2		5.8 ± 10.0	
High	4.5 ± 8.5		5.2 ± 8.9	

Family history of CVD or diabetes		0.120		0.001
No family history	4.7 ± 8.4		5.2 ± 9.3	
Positive family history	4.9 ± 7.7		5.8 ± 10.0	

Cigarette smoker		<0.001		<0.001
Never smoker	4.7 ± 8.3		5.3 ± 9.2	
Ex-smoker	4.4 ± 7.9		4.6 ± 9.1	
Current smoker	5.4 ± 8.4		6.6 ± 10.9	

Snus use		<0.001		0.139
Non-user	4.5 ± 8.0		5.4 ± 9.7	
User	5.4 ± 8.4		7.6 ± 10.0	

Physical activity		0.017		0.011
High activity	5.6 ± 9.0		5.3 ± 9.4	
Moderate activity	4.6 ± 7.9		5.4 ± 9.7	
Sedentary	4.7 ± 8.5		5.8 ± 9.8	

*P *values were determined by analysis of variance (ANOVA). All reported variables were determined at the baseline survey.

**Table 4:** Correlations between 10-year percent weight change and continuous variables by sex

Variable	Men		Women	
	r	P value	r	P value

Total cholesterol	-0.079	<0.001	-0.078	<0.001

Triglycerides	-0.064	<0.001	-0.074	<0.001

Fasting capillary glucose	-0.070	<0.001	-0.073	<0.001

2 hr postprandial glucose	-0.076	<0.001	-0.050	<0.001

Systolic blood pressure	-0.074	<0.001	-0.068	<0.001

Diastolic blood pressure	-0.097	<0.001	-0.068	<0.001

Body mass index	-0.164	<0.001	-0.149	<0.001

P values are based on Pearson correlations.

Because the univariate ANOVA analyses found no relationship between baseline self-rated health and 10-year weight change, further multivariate analyses or evaluation for confounding were deemed unnecessary and inappropriate [23].

Self-rated health at follow-up was related to weight change during the previous 10-year period (data not shown). This association was not pertinent to the aim of this study and was not evaluated further.

Univariate analyses of relationships between self-rated health with biologic and socioeconomic variables showed significant relationships in the expected directions for both men and women for all variables considered (Data not shown).

## Discussion

We found that self-rated health is unrelated to change in body weight over a period of ten years. Translated self-rated health questionnaires have been used globally with consistent findings between self-rated health and a variety of health conditions and outcomes [13,24-28]. Given the strength of the relationship of self-rated health to numerous morbidity and mortality outcomes and socioeconomic factors [10,24,29,30], we anticipated that better self-rated health would predict weight maintenance. Self-rated health at baseline was used because we hoped to find a simple tool to be used as a predictor of weight change in the clinical setting. We did not find self-rated health to be related to subsequent weight change. One possible explanation is that the impact of important determinants of weight change, physical activity and indicators of healthy eating such as intake of fruit and vegetables, despite being correlated to self-rated health, play a minor role in the concept of self-rated health. This is shown in two populations of similar age, the Whitehall II and Gazel cohort studies [14]. From a public health perspective, weight stability or maintenance over longer periods is important. In particular, maintaining a normal weight would avoid an increased morbidity and mortality burden [7]. Our results show that, at least with regard to 10-year weight change, self-rated health is not a useful predictor among middle-aged adults. Self-rated health might be related to weight changes within shorter periods of time or in other age groups, but we could not evaluate this with our data.

Univariate and descriptive analyses of the relationships between 10-year percent weight change and demographic, biologic and socioeconomic variables show significant relationships in the expected directions for both men and women. See Tables 3 and 4. The fact that most of the other considered baseline variables were correlated with weight change as expected (albeit most were only weakly correlated), provides further support for the validity of our finding. For example, higher fasting serum cholesterol and capillary glucose values were inversely correlated with 10-year weight change, ie, those with a higher cholesterol or glucose at baseline had less weight gain. This might be due to patients with hypercholesterolemia or diabetes being more attentive to recommended weight loss or maintenance. Age group also correlated inversely with weight gain, ie, older individuals had lower 10-year percent weight gain. These findings remained consistent for both socioeconomic and biologic variables.

One special group of interest is smokers, because they tend to report worse self-rated health than non-smokers [10]. In this study, weight gain was approximately one kilogram greater among smokers than non-smokers. One reason for this could have been smoking cessation during the study period (smoking at follow-up was not included in the analyses). In order to evaluate if there is a difference between smokers and non-smokers with regard to prediction of weight change based on self-reported health, the analyses were repeated and stratified for smoking category. The results did not change, ie, weight change was similar among all smokers and among all non-smokers irrespective of self-rated health at baseline. Because there was no main effect of self-rated health on weight change in the univariate analyses, multivariate analyses were mot done [23].

The finding that baseline demographic, socioeconomic and biologic variables are related to self-rated health is consistent with previously established observations and trends seen from other populations [11-14,31]. For example, age is inversely correlated with better self-rated health status (*p *< 0.001 for men and women), ie, those who are younger were more likely to report very good health. Cigarette smoking is also inversely correlated with better self-rated health (*p *< 0.001 for men and women), ie, those who smoke are less likely to report very good health. All of these relationships were in the expected directions, confirming that self-rated health assesses objective and subjective measures as previously shown in other populations.

Various cut-points have been suggested as appropriate for use in defining weight loss, weight maintenance and weight gain in population studies. We chose to illustrate our figures with less than 5% weight change (loss or gain) to assist the reader in evaluating clinically significant weight change within the population. Many studies, both intervention and observational, have found that a 5% change in weight is clinically meaningful for conditions such as hypertension and hypercholesterolemia [32-36]. Some experts have suggested less than 3% change as the best definition of weight maintenance since weight loss of approximately 2% correlates with thirst and is expected to be in the range of physiologic variation [36]. The population studied herein had an average 10-year weight change that was a 5% gain. We consider this a clinically significant change and above that expected due to day-to-day variation alone. The figures clearly show that, overall, the population is increasing in weight and there is no relationship of the weight change to self-rated health.

Others have examined the relationship of self-rated BMI to self-rated health in cross-sectional surveys and found that the extremes of weight are related to poorer self-rated health [37] or quality of life [2]. This is consistent with our finding that self-rated health at follow-up was related to weight change during the previous 10-year period, but this information is not relevant for the prediction of weight gain and cannot identify those at risk of future weight gain. Nor it is helpful in the design of interventions to maintain weight. A previous study evaluated the relationship of change in self-rated health to self-rated 6-year change in BMI category, but did not provide information about weight change in kilograms or BMI units [19]. Those results found an association between poor self-rated health and subsequent 6-year increases in BMI category. Without data on actual changes in weight or BMI, the findings are difficult to interpret and may be spurious. In addition, BMI was calculated from self-reported weight and height in that study. Problems with the validity of self-rated body mass are known and include systematic errors in weight and height [38-42].

Concordant with previous results [43], we found that in addition to older age, higher levels of traditional cardiovascular risk markers were negatively correlated with 10-year weight change. Slowing of weight gain among those with a health condition might be due to the education and influence of health care providers. Therefore, strategies to slow the obesity epidemic also need to target those who are young and apparently healthy as these individuals have the largest weight gains. This implies that broader, structural and environmental efforts are needed on the societal level [3,44].

We chose to use baseline self-rated health because we were interested in the ability to predict weight change rather than evaluate the influence of weight change on self-rated health. While some researchers have used change in BMI as the measure of weight change, we used measured percent weight change because total body weight is the most commonly used and clinically meaningful (to physicians and patients) measure currently in use [45]. We were able to calculate percent weight change because the data are from a longitudinal study. Although physicians and researchers are familiar with BMI, there are well recognized problems with using this measure for assessment of appropriate body weight for individuals. In addition, clinical studies have typically reported change in body weight as the outcome in weight loss studies intended to modify health conditions such as hypertension and hypercholesterolemia [46].

The VIP surveys comprise a very large, population-based study and use standardized questionnaires and measurement techniques. The setting of the local health clinic is familiar to most Swedes, ie, the survey is conducted in their own primary care centre, and this is expected to enhance participation. Despite the 10-year follow-up interval, the participation rate among eligible subjects was high (76%) and reflects the high credibility that the Swedish population holds for primary care. In addition, the small differences in demographic characteristics between participants and non-participants (Table 1) imply that the generalizability of the data is very strong. An analysis comparing VIP participants to those who declined to participate in an VIP initial survey also found only marginal differences between participants and non-participants with regard to social characteristics [47]. This supports our assessment that only minor social selection bias occurred in the study sample. One obvious limitation is that the study population is restricted to those aged 30-50 years at baseline and thus our results are only relevant for middle-aged populations. Only those who were alive after 10 years were included. However, due to low mortality rates in these ages we believe that this should not substantially bias the results even if those who had died might have experienced more rapid weight changes. In the current study, we used continuous variables whenever they were available in order to increase the study power. While the study was conducted in a homogenous sample of middle-aged adult Swedes, we believe the findings can be generalized to other populations. This is supported by the findings of other studies that have used self-rated health from this population [10,48,49] and have findings consistent with those of researchers in other settings and countries [18,25,50].

## Conclusions

Self-rated health did not predict 10-year weight change in a middle-aged population. We think these results are valid and stronger than those of previous studies. The population was drawn from a free-living population in Northern Sweden in which all residents aged 30-60 years were invited to participate, the number of participants is large, and both self-rated health and objective weight change were measured in a standardized way. This allows the findings to be extrapolated to other middle-aged populations with similar demographic and socioeconomic characteristics. While self-rated health remains a useful tool for predicting other outcomes, it is not able to predict weight change, whether that be weight maintenance, loss or gain over a 10-year period.

## List of abbreviations used

BMI: body mass index; CVD: cardiovascular disease; VIP: Västerbotten Intervention Programme.

## Competing interests

The authors declare that they have no competing interests.

## Authors' contributions

MN made substantial contributions to conception and study design, was involved in acquisition of data, data analyses and interpretation, and drafted and revised the manuscript. KL was involved in data interpretation and provided critical review. PLJ provided biostatistical expertice for data analyses and critical review of the manuscript. ME was involved in data interpretation and provided critical review. GL was involved in data acquisition and drop-out analyses. ANN made substantial contributions to conception and study design, was involved in data analyses and interpretation, and drafted and revised the manuscript. All authors read and approved the final manuscript.

## Pre-publication history

The pre-publication history for this paper can be accessed here:

http://www.biomedcentral.com/1471-2458/11/748/prepub
